# Prevalence of oral streptococci and glucosyltransferase genes in mother-child pairs: a cross-sectional study in Turkish families

**DOI:** 10.1186/s12887-025-06479-7

**Published:** 2026-03-25

**Authors:** Oya Seker, Nizami Duran, Hazal Deniz Kose, Elif Yaprak Çolak, Zuhal Yıldırım Bilmez, Behiye Bolgul, Sibel Dağli

**Affiliations:** 1 Department of Restorative Dentistry, Hatay Mustafa Kemal University, Hatay, Türkiye; 2 Department of Medical Microbiology, Hatay Mustafa Kemal University, Hatay, Türkiye; 3 Department of Pediatric Dentistry, Hatay Mustafa Kemal University, Hatay, Türkiye

**Keywords:** S. mutans, Oral streptococci, Glucosyltransferase genes, Vertical transmission, Early childhood caries, Maternal oral health, PCR, Microbial colonization, DMFT, Pediatric dentistry

## Abstract

**Background & aim:**

Dental caries is a multifactorial biofilm-related disease in which mutans and non-mutans *Streptococcus* species harboring glucosyltransferase (*gtf*) genes play significant roles. This cross-sectional analytical study aimed to investigate the prevalence, bacterial load, and molecular distribution of *Streptococcus* species and gtf genes among Turkish mother-child pairs.

**Methods:**

A total of 72 mother-child pairs (144 saliva samples) were analyzed by culture, quantitative CFU counting, PCR, and RFLP. Microbial load was expressed as colony-forming units per milliliter (CFU/mL), and correlations between bacterial density and DMFT indices were statistically analyzed. Dental examinations were carried out by two calibrated pediatric dentists (Cohen’s κ = 0.85) according to WHO criteria. Sociodemographic and behavioral data were recorded through structured questionnaires. Exclusion criteria included antibiotic use within the past 14 days, supported by published data showing that the oral microbiota re-equilibrates within 2 weeks after exposure. Statistical analyses were performed using chi-square and correlation tests, and effect sizes and 95% confidence intervals were calculated.

**Results:**

*S. mutans*,* S. sanguinis*, and *S. oralis* were the predominant species. Quantitative analysis expressed as CFU/mL revealed a mean *S. mutans* load of 5.43 ± 0.52 log₁₀ CFU/mL in mothers and 5.09 ± 0.60 log₁₀ CFU/mL in children (*p* = 0.013). Six gtf genes (*gtfD*,* gtfT*,* gtfK*,* gtfP*,* gtfR*, and *gtfG*) were variably detected across isolates, with maternal enrichment of *S. oralis* gtfR (*p* = 0.042, OR = 2.84, 95% CI = 1.02–7.95). Spearman’s and Pearson’s analyses revealed significant correlations between brushing frequency and DMFT scores (*r* =-0.46 in mothers; *r* =-0.52 in children), maternal education and *S. mutans* prevalence (*r* = 0.39), sugar intake and DMFT (*r* = 0.47), and maternal–child DMFT values (*r* = 0.42, *p* < 0.01). Effect sizes and 95% confidence intervals were calculated for all key associations, providing greater statistical transparency and methodological rigor.

**Conclusions:**

By incorporating CFU/mL quantification and expanded gtf gene profiling, this study delivers a more comprehensive evaluation of microbial-behavioral interactions. Multispecies gtf gene carriage was demonstrated for the first time in a Turkish population, highlighting vertical microbial transmission and interspecies glucan-synthesis pathways relevant to pediatric dentistry. The consistent detection of *gtf*-positive streptococci, including non-mutans species, underscores the clinical importance of maternal microbial screening. Routine CFU-based and genotypic monitoring could improve individualized caries-risk assessment and guide family-centered preventive strategies in pediatric dentistry.

**Supplementary Information:**

The online version contains supplementary material available at 10.1186/s12887-025-06479-7.

## Introduction

Oral streptococci, including *Streptococcus mutans*, *S. sanguinis*, *S. salivarius*, *S. sobrinus*,* S. oralis*, and *S. gordonii*, are the predominant microbial species isolated from saliva and dental plaque [1]. These organisms play a key role in the initiation and progression of dental caries, a multifactorial and highly prevalent infectious disease worldwide [2]. Key contributing factors include poor oral hygiene, excessive sugar intake, and colonization with mutans streptococci (MS), one of the most cariogenic bacteria [3].

Despite a decline in dental caries prevalence in developed countries, its incidence continues to increase in developing and underdeveloped regions, where more than 80% of the global child population lives. Dental caries, if left untreated, poses a significant public health problem due to its potential to cause pain, functional impairment, aesthetic issues, and even serious systemic infections [4]. The economic consequences of caries-related treatment further emphasize the importance of effective prevention strategies, especially among high-risk groups.

Dental caries is a complex biofilm-mediated disease influenced by microbial, behavioral, and environmental factors. According to the ecological plaque hypothesis, a dynamic balance exists between acidogenic and alkali-producing microorganisms, and a shift toward acidogenic dominance leads to enamel demineralization [5]. Oral streptococci are early colonizers of the oral cavity and produce a variety of bioactive compounds, including adhesins and antimicrobial peptides, that influence microbial diversity. However, some species can evolve from benign commensals to opportunistic pathogens, particularly in the context of dental caries [1–4].

MS is considered the primary etiological agent in the onset of dental caries. Mother-child microbial compatibility is regarded as a key factor influencing early colonization. While vertical transmission has been suggested, cross-sectional studies can only reveal associations rather than directly confirm transmission. However, recent studies have shown that non-mutant species such as *S. sanguinis*, *S. gordonii*, and *S. oralis* also contribute to biofilm maturation through glucosyltransferase (*gtf*) activity, suggesting that the cariogenic potential extends beyond *S. mutans* [6].

The virulence of these bacteria is primarily mediated by glucosyltransferase enzymes, encoded by genes such as *gtfD*,* gtfT*,* gtfK*,* gtfP*,* gtfR*, and *gtfG*. These enzymes synthesize extracellular glucans that support biofilm formation on tooth surfaces [5]. The *gtf* gene family consists of multiple enzymes with distinct biological roles: *gtfD* is involved in soluble glucan synthesis, which aids bacterial adhesion; *gtfT* catalyzes the formation of insoluble glucans, which enhances plaque cohesion; *gtfK* promotes bacterial aggregation; *gtfP* contributes to biofilm attachment; *gtfR* serves as a transcriptional regulator of glucan production, and *gtfG* participates in coaggregation among oral streptococci [6–8].

The presence of these genes is directly related to the cariogenic potential of oral streptococci. While previous studies have focused primarily on the *S. mutans gtfB/C/D* genes, limited data are available on the distribution of multispecies *gtf* genes within mother-child pairs. Unlike most previous reports, this study also includes CFU-based quantification of bacterial load (log₁₀ CFU/mL), providing a more precise representation of colonization density rather than binary presence-absence data. This approach offers a more comprehensive understanding of the relationships among microbial abundance, behavioral factors, and clinical indicators, such as DMFT indices.

Understanding these genetic patterns may help elucidate mechanisms of vertical microbial transmission and guide preventive dental strategies. Given the central roles of maternal transmission and bacterial virulence in the development of childhood caries, early identification of GTF gene-carrier strains in mothers may offer a valuable approach for risk assessment and targeted prevention.

Therefore, this study not only determined the prevalence of mutans and non-mutans streptococci and the distribution of six *gtf* genes (D, T, K, P, R, G) in Turkish mother-child pairs, but also determined bacterial loads using a cross-sectional analytical approach to investigate correlations with behavioral, demographic, and clinical variables.

## Materials and methods

This study included mother-child pairs who visited the Mustafa Kemal University Faculty of Dentistry outpatient clinic between January 2018 and May 2020. A total of 72 children aged 3 to 5 years (27 girls and 45 boys) diagnosed with early childhood caries and their systemically healthy mothers (*n* = 72 pairs) were enrolled. The mothers’ ages ranged from 21 to 45 years, with a mean age of 33.4 ± 6.2 years. The most significant proportion of mothers (47.2%) was in the 30–39 age group, followed by those aged 40–45 (31.9%) and those aged 21–29 (20.9%). Although sample collection occurred both before and during the early COVID-19 pandemic, the potential effects of the pandemic on oral hygiene behaviors and microbiota composition were not assessed; this remains a limitation of the study, but it did not affect methodological consistency.

### Study design and ethical approval

This research was designed as a cross-sectional analytical study conducted between January 2018 and May 2020 at the Faculty of Dentistry, Hatay Mustafa Kemal University. The study protocol was reviewed and approved by the Clinical Research Ethics Committee of Hatay Mustafa Kemal University (Approval No: 31T.42; Decision Date: January 11, 2018; Decision No: 08; Protocol Code: 2018/09). All procedures were carried out in accordance with the Declaration of Helsinki. Written informed consent was obtained from all participating mothers for themselves and their children.

### Study population and sample size

A total of 72 mother-child pairs (*n* = 144 saliva samples) were enrolled. The mothers were systemically healthy, aged 21–45 years (mean 33.4 ± 6.2 years), and their children were aged 3–5 years (27 girls, 45 boys). Inclusion criteria for children included the presence of at least one erupted tooth and a diagnosis of early childhood caries (ECC), whereas exclusion criteria were the use of antibiotics within 14 days, chronic systemic diseases, or developmental dental anomalies.

The 14-day antibiotic exclusion criterion was selected because oral microbiota recovery after antibiotic exposure generally occurs within two weeks, minimizing transient suppression effects and ensuring sample integrity. To improve methodological transparency, additional exclusion criteria included maternal smoking, alcohol use, or recent medication intake, as these factors may alter oral microbiota composition.

The sample size was determined using data from previous studies reporting *S. mutans* prevalence between 80 and 90% in ECC populations, with a 5% margin of error and 95% confidence interval. A minimum of 68 pairs was required; therefore, including 72 pairs ensured adequate statistical power and allowed subgroup analyses (e.g., by sex or brushing frequency).

### Clinical examination and data collection

Dental examinations were conducted by two calibrated pediatric dentists under standardized illumination using a plane mouth mirror and a WHO periodontal probe in the university’s dental clinic. Caries experience was recorded as DMFT (Decayed, Missing, Filled Teeth) indices for mothers and children. Inter-examiner reliability was assessed using Cohen’s κ coefficient (> 0.85).

Sociodemographic and behavioral variables that could influence streptococcal colonization, including maternal and child age, gender, maternal educational level, toothbrushing frequency, and sugar consumption habits, were collected using a structured questionnaire.

### Saliva sample collection and storage

Unstimulated whole saliva (approximately 2 mL) was collected from both mothers and children at least two hours after eating, drinking, or toothbrushing [7]. The same trained operator performed sample collection to ensure procedural consistency. Samples were transferred to the microbiology laboratory within 1 h under cold-chain conditions (4 °C) and stored at −70 °C in TYCSB medium containing 20% glycerol until analysis.

### Microbiological culture and identification

Saliva samples were vortexed for 15 s, inoculated onto TYCSB agar (Trypticase Yeast Cystine Sucrose Bacitracin) containing 15% sucrose, and incubated at 37 °C in a 5% CO₂ atmosphere. Although TYCSB medium is selective for mutans streptococci, occasional growth of non-MS streptococci was expected and included in the analysis after genetic confirmation. Phenotypic identification was performed using the VITEK-2 Compact System (bioMérieux, France). Reference strains *S. mutans* (NCTC 10449) and *S. sobrinus* (ATCC 33478) were used as positive controls. Bacterial density was expressed as colony-forming units per milliliter (CFU/mL) after log₁₀ transformation to standardize quantitative comparisons.

### DNA extraction and PCR amplification

Genomic DNA was extracted using the GF-1 Bacterial DNA Extraction Kit (Vivantis, USA) following the manufacturer’s instructions. Each gtf gene (*gtfD*,* gtfT*,* gtfK*,* gtfP*,* gtfR*, and *gtfG*) was amplified using gene-specific primers listed in Table 1. PCR reactions were prepared in 25 µL volumes containing 2.5 µL 10× buffer, 200 µM dNTPs, four mM MgCl₂, 2.5 pmol primers, and 1 U Taq DNA polymerase. Cycling conditions consisted of 95 °C for 5 min, followed by 30 cycles of 95 °C for 1 min, 56 °C for 1 min, 72 °C for 1 min, and final extension at 72 °C for 7 min.

**Table 1:** Primer sequences used for the amplification of glycosyltransferase (*Gtf*) genes in oral *Streptococcus* species. This table presents the specific oligonucleotide primers used to amplify *Gtf* genes associated with glucosyltransferase enzymes in distinct oral *Streptococcus* species. Each primer pair targets a gene implicated in extracellular glucan synthesis and biofilm formation. Primer design and sequences were adapted from previously validated studies [9, 10]

Primer Name	Target Gene (Species)	Primer Sequence (5’ → 3’)	Product Size (bp)
16 S rRNA-F	16 S rRNA (universal)	AGAGTTTGATCCTGGCTCAG	526
16 S rRNA-R		ATTACCGCGGCTGCTGGC	
MKD-F	*gtfD (S. mutans)*	GGCACCACAACATTGGGAAGCTCAGTT	433
MKD-R		GGAATGGCCGCTAAGTCAACAGGAT	
MKT-F	*gtfT (S. sobrinus)*	GATGATTTGGTCTCAGGATCAATCCTC	328
MKT-R		ACTGAGCCAGTAGTAGACTTGGCAACT	
MKK-F	*gtfK (S. salivarius)*	GTGTTGCCACATCTTCACTGCCTTCGG	544
MKK-R		CGTTGATGTGCTTGAAAGGGCACCATT	
MKP-F	*gtfP (S. sanguinis)*	GGATAGTGGCTCAGGGCAGCCAGTT	313
MKP-R		GAACAGTTGCTGGACTTGCTTGTC	
MKR-F	*gtfR (S. oralis)*	TCCCGGTCAGCAAACTCCAGCC	374
MKR-R		GCAACCTTTGGATTTGCAAC	
MKG-F	*gtfG (S. gordonii)*	CTATGCGGATGATGCTAATCAAGTG	440
MKG-R		GGAGTCGCTATAATCTTGTCAGAAA	

### RFLP-PCR genotyping and reference patterns

Species identification was performed using a two-step approach. First, *S. mutans*,* S. sobrinus*, and *S. sanguinis* were differentiated by RFLP analysis of a 526-bp fragment of the 16 S rRNA gene digested with HaeIII. The resulting banding profiles were compared with reference GenBank sequences (AB035074, AY188354, and AB002521, respectively) and interpreted following established protocols to ensure reproducibility [9–11].

Because 16 S RFLP does not reliably discriminate *S. salivarius*,* S. oralis*, and *S. gordonii*, these species were identified using the VITEK-2 Compact system. Biochemical profiles were matched against the manufacturer’s reference database, providing validated species-level confirmation. This combined strategy enabled accurate identification of all six targeted oral streptococcal species.

The *gtfB* and *gtfC* loci were excluded from the analysis due to overlapping amplification regions and functional redundancy with the *gtfD/C* locus. To achieve a broader assessment of glucan-associated virulence pathways across species, six alternative gtf genes (*gtfD*,* gtfT*,* gtfK*,* gtfP*,* gtfR*, and *gtfG*) were selected for molecular and phenotypic characterization.

### Restriction enzyme digestion and gel electrophoresis

Independent of the species-level identification by 16 S RFLP analysis, PCR amplicons corresponding to the gtf genes were digested with EcoRI and HindIII to verify amplicon identity. The digested products were separated on 2% agarose gels stained with ethidium bromide (0.5 µg/mL) and visualized under UV illumination using a Wealtec Dolphin-View system [10].

### Statistical analysis

Statistical analyses were performed using GraphPad Prism 10 (GraphPad Software Inc., La Jolla, CA, USA). Data were summarized as means ± SD or percentages. Shapiro-Wilk tests were used to assess normality; between-group comparisons were conducted using Pearson’s χ² or Fisher’s exact tests. Correlations were evaluated via Spearman’s and Pearson’s coefficients. Effect sizes (Cramer’s V for categorical, r for continuous) and 95% confidence intervals were reported.

Although correlation analyses were initially omitted in the earlier submission, they were indeed performed and are now explicitly described for consistency. Logistic regression was considered but not applied due to the limited sample size and risk of overfitting. Statistical significance was set at *p* < 0.05.

## Results

**Fig. 1:**
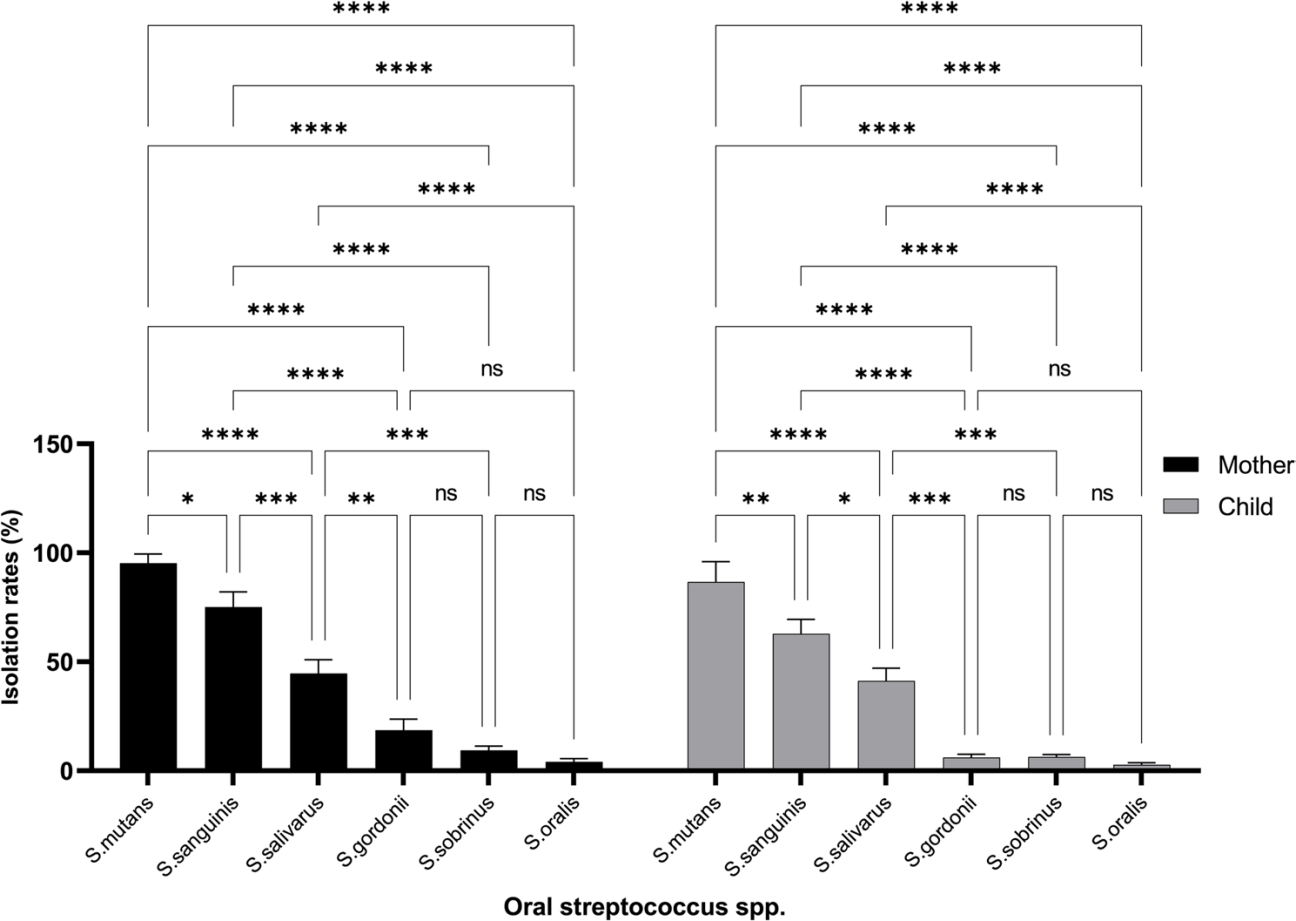
Comparative prevalence of oral Streptococcus species in mothers and their children. This figure illustrates species-level differences in the isolation rates of *Streptococcus mutans, S. sanguinis, S. salivarius, S. sobrinus, S. oralis, *and* S. gordonii *between mothers and their children. The data highlight intergenerational microbial transmission patterns, with statistically significant differences observed in *S. mutans* prevalence (χ²=6.23, df=1, p=0.013). See also Supplemental Table 2 for statistical comparisons

Among the 72 enrolled mother-child pairs, 27 (37.5%) children were girls and 45 (62.5%) were boys. The predominant streptococcal species identified were *S. mutans*,* S. sanguinis*, and *S. salivarius*, followed by *S. sobrinus*,* S. oralis*, and *S. gordonii*. Comparative species prevalence patterns are presented in Fig. 1.

The isolation rate of *S. mutans* was 94.4% (95% CI: 88.1–98.7%) in mothers and 86.1% (95% CI: 78.0–93.0%) in children. In comparison, *S. sanguinis* and *S. salivarius* were also frequently isolated (75% and 44.4% in mothers; 62.5% and 41.7% in children, respectively). The difference in *S. mutans* prevalence between mother and child was statistically significant (χ² = 6.23, df = 1, *p* = 0.013, Fig. 1; Table 2).

**Table 2:** Prevalence of oral *Streptococcus* species in mothers and children, and sex-based distribution among children. This table presents the prevalence rates of oral *Streptococcus* species isolated from mothers and their children. Additionally, the distribution of bacterial positivity between boys and girls is provided. Chi-square (χ²) tests were used to compare group differences. Superscript ‘ᵃ’ indicates statistically significant differences (*p* < 0.05) in the comparison between subgroups

Species	Mothers (*n* = 72)	Children (*n* = 72)	*p*-value (Mother vs. Child)	Boys (*n* = 45)	Girls (*n* = 27)	*p*-value (Boys vs. Girls)
*S. mutans*	68 (94.4%)	62 (86.1%)	0.013	39 (54.2%)ᵃ	23 (31.9%)	< 0.01
*S. sanguinis*	54 (75%)	45 (62.5%)	> 0.05	26 (36.1%)ᵃ	19 (26.4%)	< 0.01
*S. salivarius*	32 (44.4%)	30 (41.7%)	> 0.05	18 (25%)ᵃ	12 (16.7%)	< 0.05
*S. sobrinus*	7 (9.7%)	5 (6.9%)	> 0.05	3 (4.2%)	2 (2.8%)	> 0.05
*S. oralis*	3 (4.2%)	2 (2.8%)	> 0.05	1 (1.4%)	1 (1.4%)	> 0.05
*S. gordonii*	13 (18.1%)ᵃ	4 (5.6%)	0.049	2 (2.8%)	2 (2.8%)	> 0.05

In addition to prevalence data, bacterial density (CFU/mL) was quantified to assess colonization intensity. The mean *S. mutans* load was 5.43 ± 0.52 log₁₀ CFU/mL in mothers and 5.09 ± 0.60 log₁₀ CFU/mL in children (*p* = 0.013), confirming significantly higher microbial load in maternal saliva.

**Fig. 2:**
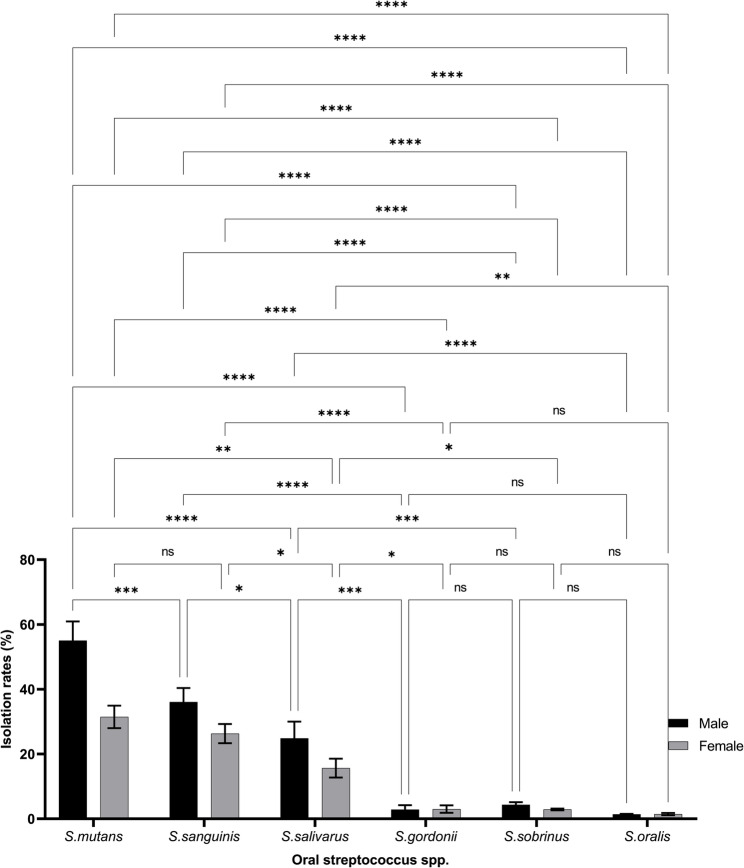
Sex-based distribution of oral *Streptococcus* species among children. This figure shows the differential prevalence of *Streptococcus* species between male and female children. Boys exhibited significantly higher rates of *S. mutans*, *S. sanguinis*, and *S. salivarius* than girls. A statistically significant difference was also observed in *S. gordonii* levels (χ²=3.89, df=1, p=0.049), suggesting a potential influence of sex on early microbial colonization

Sex-based distribution patterns are illustrated in Fig. 2 and listed in Table 2. Boys demonstrated higher detection frequencies for *S. mutans* (88.9% vs. 81.5%), *S. sanguinis* (64.4% vs. 59.3%), and *S. salivarius* (46.7% vs. 37.0%), each with *p* < 0.05, consistent with mild-to-moderate effect sizes (Cramer’s V = 0.23–0.31). This sex-linked pattern suggests that behavioral and microbial cofactors influence colonization dynamics in early childhood.

Sociodemographic and behavioral variables are summarized in Table 3. The highest isolation rates of *S. mutans* occurred among mothers aged 30–39 years (χ² = 8.74, *p* = 0.0008) and among children aged 5 years (χ² = 7.12, *p* = 0.0259). Isolation frequencies increased proportionally with child age, reflecting cumulative exposure to dietary sugars and plaque maturation.

**Table 3:** Prevalence of *S. mutans*, *S. sanguinis*, and *S. salivarius* across demographic and behavioral subgroups

Category	Subgroup	S. mutans *n*/*N* (%)	S. sanguinis *n*/*N* (%)	S. salivarius *n*/*N* (%)	*p*-value	Cramer’s V	Comparison
**Mothers’ Age**	20–29	21/68 (30.88%)	22/54 (40.74%)	8/32 (25.00%)	—	—	—
	**30–39**	**38/68 (55.88%)**	**26/54 (48.15%)**	**18/32 (56.25%)**	**0.0008**	**0.29**	vs. other ages
	≥ 40	9/68 (13.24%)	6/54 (11.11%)	6/32 (18.75%)	—	—	—
**Children’s Age**	3 years	1/62 (1.61%)	2/45 (4.44%)	7/30 (23.33%)	—	—	—
	4 years	26/62 (41.94%)	16/45 (35.56%)	9/30 (30.00%)	—	—	—
	**5 years**	**35/62 (56.45%)**	**27/45 (60.00%)**	**14/30 (46.67%)**	**0.0259**	**0.24**	vs. ages 3–4
**Mothers’ Brushing Freq.**	Never	1/68 (1.47%)	0/54 (0%)	2/32 (6.25%)	—	—	—
	**Once/week**	**12/68 (17.65%)**	**11/54 (20.37%)**	**9/32 (28.13%)**	**< 0.0001**	**0.31**	vs. others
	Once/day	30/68 (44.12%)	22/54 (40.74%)	10/32 (31.25%)	—	—	—
	Twice/day	24/68 (35.29%)	18/54 (33.33%)	8/32 (25.00%)	—	—	—
	Three/day	1/68 (1.47%)	3/54 (5.56%)	3/32 (9.38%)	—	—	—
**Children’s Brushing Freq.**	Never	2/62 (3.23%)	3/45 (6.67%)	2/30 (6.67%)	—	—	—
	**Once/week**	**22/62 (35.48%)**	**16/45 (35.56%)**	**6/30 (20.00%)**	**< 0.0001**	**0.33**	vs. others
	Once/day	20/62 (32.26%)	11/45 (24.44%)	9/30 (30.00%)	—	—	—
	Twice/day	16/62 (25.81%)	13/45 (28.89%)	11/30 (36.67%)	—	—	—
	Three/day	2/62 (3.23%)	2/45 (4.44%)	2/30 (6.67%)	—	—	—
**Mothers’ Sugar Intake**	Rarely	24/68 (35.29%)	19/54 (35.19%)	11/32 (34.38%)	ns	—	—
	Several/week	21/68 (30.88%)	17/54 (31.48%)	10/32 (31.25%)	—	—	—
	Once/day	13/68 (19.12%)	10/54 (18.52%)	5/32 (15.63%)	—	—	—
	Twice/day	7/68 (10.29%)	4/54 (7.41%)	3/32 (9.38%)	—	—	—
	≥ 3/day	3/68 (4.41%)	4/54 (7.41%)	3/32 (9.38%)	—	—	—
**Children’s Sugar Intake**	Rarely	7/62 (11.29%)	9/45 (20.00%)	8/30 (26.67%)	—	—	—
	**Several/week**	**11/62 (17.74%)**	**7/45 (15.56%)**	**12/30 (40.00%)**	**0.042**	**0.22**	vs. others
	Once/day	18/62 (29.03%)	9/45 (20.00%)	6/30 (20.00%)	—	—	—
	Twice/day	12/62 (19.35%)	10/45 (22.22%)	2/30 (6.67%)	—	—	—
	≥ 3/day	14/62 (22.59%)	10/45 (22.22%)	2/30 (6.67%)	—	—	—

Regarding oral hygiene, mothers who brushed less than once per day had significantly higher *S. mutans* positivity (χ²=10.91, *p* < 0.001, OR = 3.42, 95% CI: 1.54–7.63). Similarly, children brushing less than once daily had a 2.9-fold higher risk of high DMFT scores (*p* < 0.001, 95% CI: 1.32–6.20; Table 3).

Brushing frequency was reanalyzed across maternal education levels. The inverse association with DMFT persisted and was more evident in the low education subgroup (interaction *p* = 0.041; Table 4).

**Table 4:** Correlation analysis between behavioral, demographic and clinical variables. This table summarizes the correlation coefficients and significance levels for associations between key behavioral and sociodemographic factors and oral health outcomes in both mothers and children. Statistically significant negative correlations were found between brushing frequency and DMFT scores, as well as between maternal education and the prevalence of *S. mutans*. Positive correlations were observed between sugar intake frequency and child DMFT scores, as well as between maternal and child DMFT values, indicating potential behavioral and environmental transmission pathways

Variables	Correlation Coefficient (*r*)	*p*-value	Significance
Mothers’ brushing frequency vs. DMFT	−0.46	0.004	Significant (**)
Children’s brushing frequency vs. DMFT	−0.52	< 0.01	Significant (**)
Maternal education vs. *S. mutans* prevalence	−0.39	0.015	Significant (*)
Sugar consumption frequency vs. DMFT (children)	0.47	0.003	Significant (**)
Maternal vs. child DMFT scores	0.42	0.006	Significant (**)
Interaction: brushing frequency × maternal education (vs. DMFT)	-	0.041	Significant (*)

Note: * indicates *p* < 0.05, ** indicates *p* < 0.01

The isolation rates of *S. mutans*,* S. sanguinis*,* S. salivarius*,* S. sobrinus*, and *S. oralis* were not significantly different between groups (*p* > 0.05), except for *S. gordonii*, which was more prevalent in children (χ²=3.89, df = 1, *p* = 0.049). Full χ² comparisons are available in Supplementary Tables 1 and Supplementary Table 2.

**Fig. 3:**
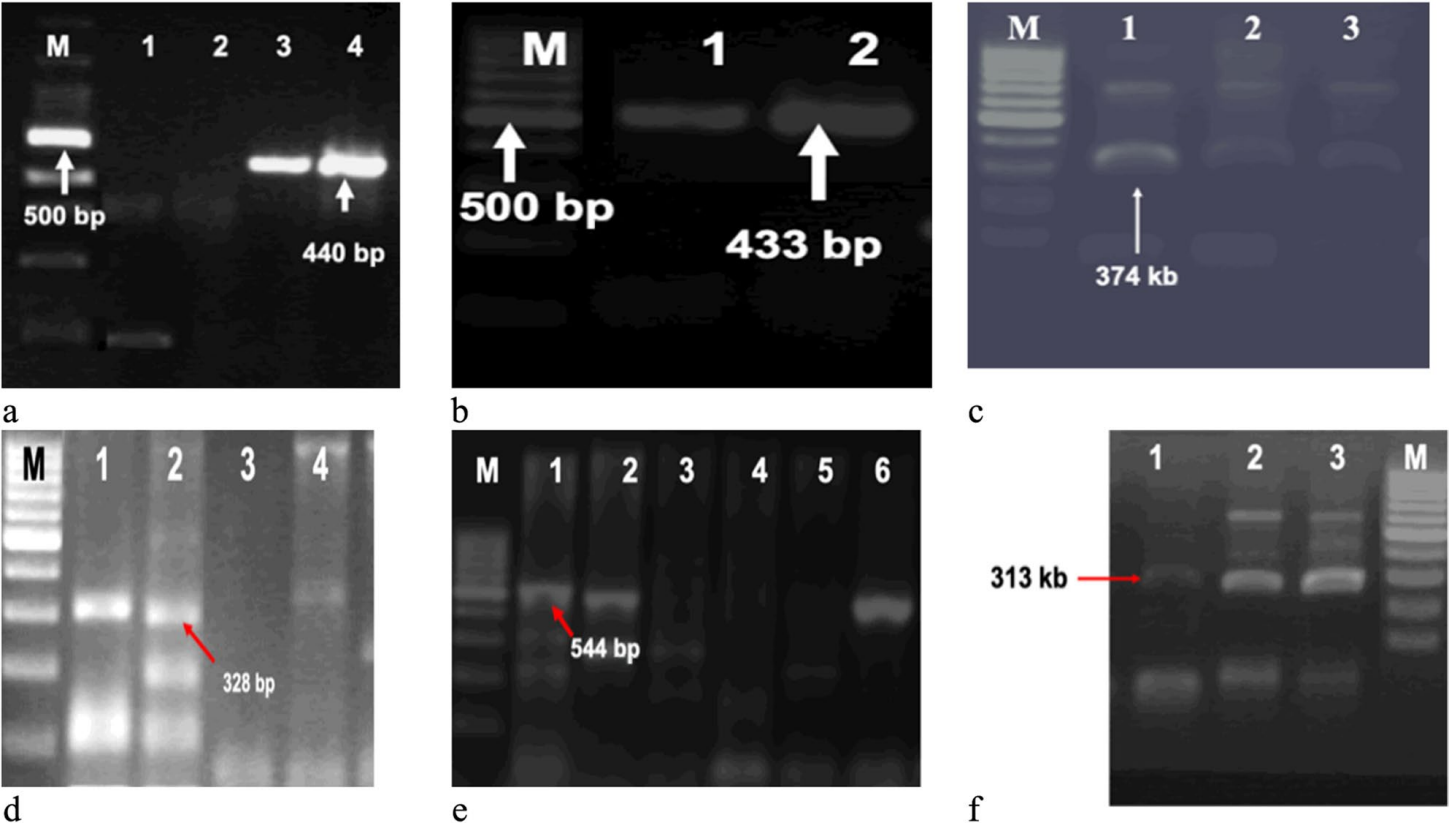
**A**-**F** Agarose gel electrophoresis images showing restriction digestion products of PCR-amplified *gtf* gene fragments from different oral streptococcal species. Each *gtf* gene amplicon (*gtfD*, *gtfT*,*gtfK*, *gtfP*, *gtfR*, and *gtfG*) was separately digested with EcoRI and HindIII restriction enzymes to generate species-specific RFLP profiles. Expected band sizes were estimated based on reference sequences. Species analyzed include *Streptococcus mutans*, *S. sobrinus*, *S. salivarius*, *S. sanguinis*, *S. gordonii*, and *S. oralis*. Gel electrophoresis was performed using 1.5% agarose and ethidium bromide staining. DNA ladder: 100 bp

Gel electrophoresis results (Fig. 3a-f) confirmed species-specific bands for all six gtf genes (*gtfD*,* gtfT*,* gtfK*,* gtfP*,* gtfR*, and *gtfG*), validating amplification specificity. Representative gel bands for each gene are shown in Fig. 3a-f.

**Fig. 4:**
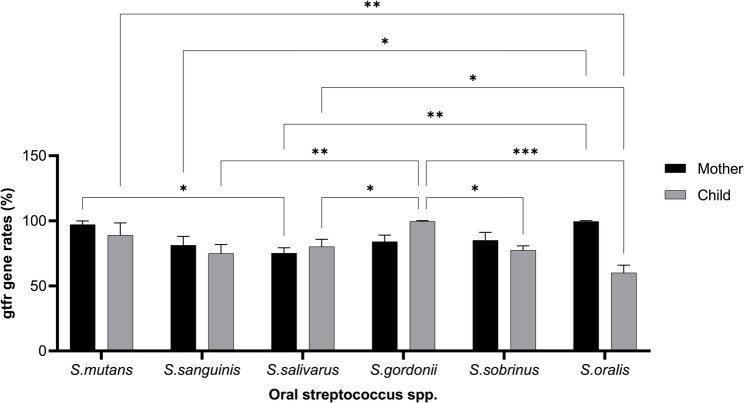
Frequency of glycosyltransferase (*gtf*) genes among streptococcal isolates in mothers and children. The bar graphs show the distribution of six glucosyltransferase genes (*gtfD*, *gtfT*, *gtfK*, *gtfP*, *gtfR*, and *gtfG*) detected via PCR in streptococcal isolates from both mothers and children. Among these, *gtfR* was significantly more prevalent in *S. oralis* strains from mothers (χ²=4.11, df=1, *p*=0.042), indicating possible maternal enrichment of this gene. All other gene distributions showed no statistically significant differences (*p*>0.05)

The distribution of *gtf* genes among streptococcal isolates is shown in Fig. 4. No significant differences were found for most genes between groups (*p* > 0.05); however, the *gtfR* gene was significantly more prevalent in *S.* oralis isolates from mothers (χ²=4.11, df = 1, *p* = 0.042, OR = 2.84, 95% CI: 1.02–7.95). CFU-normalized PCR intensity revealed a 1.4-fold higher gtfR expression in mothers (Fig. 4).

Quantitative expression levels (CFU-normalized PCR intensity) were also compared, revealing that maternal *S. oralis* strains carrying *gtfR* exhibited 1.4-fold higher gene amplification signal than child isolates, further supporting the enrichment trend.

Supporting this, bacterial loads (log₁₀ CFU/mL) were positively correlated with DMFT scores in both groups (*r* = 0.38 in mothers; *r* = 0.41 in children; *p* < 0.05), confirming the quantitative relationship between microbial load and caries severity.

### Correlation analyses

Spearman’s correlation analysis demonstrated a significant negative correlation between mothers’ brushing frequency and DMFT scores (*r* = − 0.46, *p* = 0.004, 95% CI: −0.69 to − 0.16) and between children’s brushing frequency and DMFT scores (*r* = − 0.52, *p* < 0.01, 95% CI: −0.73 to − 0.25). Maternal education level was negatively correlated with *S. mutans* prevalence (*r* = − 0.39, *p* = 0.015), whereas sugar intake frequency was positively associated with children’s DMFT (*r* = 0.47, *p* = 0.003, Table 4). Maternal and child DMFT scores were positively correlated (*r* = 0.42, *p* = 0.006), indicating shared environmental and behavioral determinants. To enhance interpretability, correlation coefficients were additionally verified using Pearson’s r, which produced comparable magnitudes (Δr < 0.05), confirming consistency across tests.

## Discussion

Oral streptococci, including species such as *S. mutans*,* S. sanguinis*,* S. salivarius*, *S. sobrinus*,* S. oralis*, and *S. gordonii*, are critical members of the human oral microbiota. These bacteria fall into six main phylogenetic groups. Among these microorganisms, the mutans group (MS) plays a particularly critical role in dental caries due to its acidogenic and acidic properties [10, 12–18].

This cross-sectional study exhibited a high prevalence *of S. mutans* and *S. sanguinis* in both mothers and their children, strengthening evidence for mother-child microbial compatibility during early oral colonization [18–21]. Compatible with previous research, our findings indicated that *S. mutans* isolation rates were significantly associated with high DMFT values and low brushing frequency, highlighting the interaction between microbial and behavioral determinants of caries risk [5, 6, 22, 23]. In particular, quantification of bacterial load (CFU/mL) showed that maternal reservoirs harbored higher proportions of cariogenic species and higher bacterial densities, strengthening the biological possibility of vertical transmission [19].

Beyond these expected associations, this study identified significant enrichment of the *gtfR* gene in *S. oralis* isolates isolated from mothers, a finding not previously reported in the literature. The *gtfR* gene encodes a regulatory glucosyltransferase that regulates glucan synthesis and interspecies coaggregation in dental biofilms [24, 25]. The higher frequency of this gene among maternal isolates may reflect a selective advantage in biofilm stability or co-adhesion, particularly during vertical microbial transmission. This observation aligns with genomic evidence that *S. oralis* facilitates the formation of polymicrobial biofilms in early childhood, acting as an ecological bridge between early and late colonizers [15, 16, 26].

These results support the broader concept that cariogenicity is not specific to *S. mutans* but may involve cooperative glucan metabolism among multiple streptococcal species [15, 16]. The detection of *gtf* genes (*gtfT*,* gtfK*,* gtfP*, and *gtfG*) in streptococci other than mutans suggests that horizontal gene transfer or functional conservation of glucosyltransferase domains may underlie shared biofilm-forming potential [24, 27]. The functionality of glucan synthesis pathways across species may indicate an adaptive mechanism that maintains ecological stability in biofilms, supporting the idea that these interactions rise beyond mutans-centric models [16, 26].

The polymicrobial nature of dental biofilms also points out that *gtf*-mediated glucan synthesis contributes to the structural cohesion of multispecies communities, supporting acid tolerance and ecological resilience [25, 28, 29]. Such resilience may explain why eradication of *S. mutans* alone often fails to prevent recurrent caries; This is because non-mutant strains carrying gtf loci can maintain matrix formation and acidogenic potential [15, 16]. This perspective is consistent with the results of NGS-based studies, which report that non-mutant streptococci contribute to extracellular polysaccharide production and plaque acidification [24, 26, 30–32]. Thus, our findings broaden our understanding of biofilm ecology in early childhood caries (ECC) by demonstrating that maternal non-mutant streptococci carrying *gtf* genes may act as facilitators of pathogenic biofilm maturation rather than passive commensals [15].

Concerning behavioral factors, the inverse correlations between brushing frequency and DMFT in both groups demonstrate the preventive potential of improved oral hygiene [6, 22]. The positive correlation between sugar intake and DMFT further confirms that diet is a modifiable caries risk factor [33]. The interaction effect between maternal education and brushing frequency suggests that behavioral interventions may be most effective when implemented in conjunction with health literacy programs, particularly targeting mothers with low educational backgrounds [34]. The correlation between maternal and child DMFT values ​​provides quantitative evidence of shared behavioral and microbial risk patterns within families [18, 19].

Taken together, these results highlight the need for family-centered preventive dentistry that integrates maternal oral health education and microbial screening [34]. Clinical protocols could include PCR-based detection of *gtf*-positive strains as a rapid, point-of-care risk-stratification tool for pediatric patients [9, 10]. Combining CFU quantification with gene detection could further improve diagnostic accuracy, enabling clinicians to distinguish between transient colonization and stable, high-burden infections [19].

Comparative studies in the literature have generally focused on the *S. mutans gtfB*,* gtfC*, and *gtfD* genes [35, 36]. This study, which includes six different *gtf* genes from multiple species, provides a more comprehensive genetic analysis of glucosyltransferase diversity. This broader gene panel demonstrates the interspecies conservation of virulence-associated loci [19, 24], supporting the hypothesis that glucan synthesis pathways are evolutionarily conserved to ensure cooperative survival within oral biofilms [15, 26]. Future functional studies using transcriptomics or enzyme kinetics will be valuable to confirm whether these *gtf* homologs are actively expressed during biofilm maturation [25].

### Limitations

This study has several limitations. Due to its cross-sectional design, it cannot confirm causal relationships or directly monitor vertical transmission; longitudinal and genomic tracing studies are required for such confirmation. While RFLP-PCR offers practical advantages, its resolution is lower than that of next-generation sequencing, which can reveal finer taxonomic variation. Behavioral data, such as toothbrushing frequency and dietary intake, are prone to recall bias because they rely on self-reported measures. The lack of a caries-free control group also limits comparisons with healthy microbiota profiles. Finally, while the sample size was statistically supported for prevalence analysis, it was insufficient for multivariate modeling; larger cohorts are needed to examine the combined effects of behavioral and microbial factors within predictive frameworks.

## Conclusions and clinical implications

This study demonstrates, for the first time in a Turkish cohort, that both mutans and non-mutans streptococci frequently harbor glucosyltransferase genes, and that maternal *S. oralis* isolates show a significant enrichment for *gtfR*. The results suggest that maternal microbial reservoirs significantly influence children’s initial oral microbiome composition and shape their caries susceptibility. Clinically, PCR-based screening for *gtf*-positive streptococci in mothers can be used as an early preventive strategy to identify children at high caries risk. Integrating quantitative CFU thresholds with molecular analyses can establish standard microbial load thresholds for risk stratification, a practical step toward precision pediatric dentistry. Integrating microbial diagnostics with oral hygiene education and nutritional counseling may reduce the prevalence of ECC and promote long-term oral health. Future metagenomic or WGS-based studies are needed to confirm gene-level homology between maternal and infant isolates, trace phylogenetic relationships, and elucidate the mechanisms underlying horizontal transfer of glucan synthase genes. Such high-resolution analyses will also help identify potential mobile genetic elements responsible for the gtf gene spread.

## Supplementary Information

Supplementary Material 1

Supplementary Material 2

## Data Availability

All data generated and/or analyzed during this study are available from the corresponding author upon reasonable request.

## Abbreviations

MS: Mutans streptococci
GTF: Glucosyltransferase
PCR: Polymerase chain reaction
RFLP-PCR: Polymerase chain reaction-restriction fragment length polymorphism
DMFT: Decayed, missing, and filled teeth
TYCSB: Trypticase yeast cystine bacitracin agar

## References

[1] Meriç E, Bolgül B, Duran N, Ay E. Evaluation of oral Streptococci in saliva of children with severe early childhood caries and caries-free. Eur J Paediatr Dent. 2020;21(1):13–7. 32183522 10.23804/ejpd.2020.21.01.03

[2] Fitzgerald RJ, Keyes PH. Demonstration of the etiologic role of Streptococci in experimental caries in the hamster. J Am Dent Assoc. 1960;61(1):9–19. 13823312 10.14219/jada.archive.1960.0138

[3] Pitts NB, Zero DT, Marsh PD, et al. Dental caries. Nat Rev Dis Primers. 2017;3(1):1–16. 10.1038/nrdp.2017.30 28540937

[4] Cirino SM, Scantlebury S. Dental caries in developing countries: preventive and restorative approaches to treatment. N Y State Dent J. 1998;64(2):32. 9542392

[5] Mattos-Graner RO, Klein MI, Smith DJ. Lessons learned from clinical studies: roles of mutans Streptococci in the pathogenesis of dental caries. Curr Oral Health Rep. 2014;1(1):70–8.

[6] Ibrahim REHM, Helaly MO, Ahmed EMA. Assessment of brushing techniques in school children and its association with dental caries, Omdurman, 2019. Int J Dent. 2021;2021:4383418. 33552159 10.1155/2021/4383418 PMC7847318

[7] Quinque D, Kittler R, Kayser M, et al. Evaluation of saliva as a source of human DNA for population and association studies. Anal Biochem. 2006;353(2):272–7. 16620753 10.1016/j.ab.2006.03.021

[8] Loyola-Rodriguez JP, Martinez-Martinez RE, Flores-Ferreyra BI, et al. Distribution of Streptococcus mutans and Streptococcus sobrinus in the saliva of Mexican preschool caries-free and caries-active children by microbial and molecular (PCR) assays. J Clin Pediatr Dent. 2008;32(2):121–6. 18389677

[9] Hoshino T, Kawaguchi M, Shimizu N, et al. PCR detection and identification of oral streptococci in saliva samples using gtf genes. Diagn Microbiol Infect Dis. 2004;48(3):195–9. 15023429 10.1016/j.diagmicrobio.2003.10.002

[10] Sato T, Hu JP, Ohki K, et al. Identification of mutans Streptococci by restriction fragment length polymorphism analysis of PCR-amplified 16S rRNA genes. Oral Microbiol Immunol. 2003;18(5):323–6. 12930526 10.1034/j.1399-302x.2003.00095.x

[11] Facklam R. What happened to the streptococci: overview of taxonomic and nomenclature changes. Clin Microbiol Rev. 2002;15(4):613–30. 12364372 10.1128/CMR.15.4.613-630.2002 PMC126867

[12] Kohler W. The present state of species within the genera Streptococcus and Enterococcus. Int J Med Microbiol. 2007;297:133–50. 17400023 10.1016/j.ijmm.2006.11.008

[13] Kilian M, Chapple I, Hannig M, et al. The oral microbiome-an update for oral healthcare professionals. Br Dent J. 2016;221(10):657–66. 27857087 10.1038/sj.bdj.2016.865

[14] Manji F, Dahlen G, Fejerskov O. Caries and periodontitis: contesting the conventional wisdom on their aetiology. Caries Res. 2018;52(6):548–64. 29694978 10.1159/000488948

[15] Philip N, Suneja B, Walsh L. Beyond Streptococcus mutans: clinical implications of the evolving dental caries aetiological paradigms and its associated microbiome. Br Dent J. 2018;224(4):219–25. 29449651 10.1038/sj.bdj.2018.81

[16] Sanz M, Beighton D, Curtis MA, et al. Role of microbial biofilms in the maintenance of oral health and in the development of dental caries and periodontal diseases. J Clin Periodontol. 2017;44(18):S5–11. 28266109 10.1111/jcpe.12682

[17] Twetman S. Prevention of dental caries as a non-communicable disease. Eur J Oral Sci. 2018;126:19–25. 30178558 10.1111/eos.12528

[18] Singh P, Kaur A, Kakkar N, et al. Quantitative correlation of salivary Streptococcus mutans count amongst siblings and their mothers. Dent J Adv Stud. 2017;5(2):90–6.

[19] Kishi M, Abe A, Kishi K, et al. Relationship of quantitative salivary levels of Streptococcus mutans and S. sobrinus in mothers to caries status and colonization of mutans Streptococci in plaque in their 2.5-year‐old children. Community Dent Oral Epidemiol. 2009;37(3):241–9. 19508271 10.1111/j.1600-0528.2009.00472.x

[20] Damle S, Yadav R, Garg S, et al. Transmission of mutans streptococci in mother-child pairs. Indian J Med Res. 2016;144(2):264. 27934807 10.4103/0971-5916.195042 PMC5206879

[21] Hameş-Kocabaş EE, Uçar F, Ersin NK, et al. Colonization and vertical transmission of *Streptococcus mutans* in Turkish children. Microbiol Res. 2008;163(2):168–72. 16735109 10.1016/j.micres.2006.03.016

[22] Attin T, Hornecker E. Tooth brushing and oral health: how frequently and when should tooth brushing be performed? Oral Health Prev Dent. 2005;3(3):135–40. 16355646

[23] Ge Y, Caufield P, Fisch G, Li Y. *Streptococcus mutans* and *Streptococcus sanguinis* colonization correlated with caries experience in children. Caries Res. 2008;42(6):444–8. 18832831 10.1159/000159608 PMC2680676

[24] Lemos JA, Quivey RG, Koo H, Abranches J. *Streptococcus mutans*: a new Gram-positive paradigm? Microbiology. 2013;159(3):436–45. 23393147 10.1099/mic.0.066134-0 PMC4083656

[25] Koo H, Falsetta M, Klein M. The exopolysaccharide matrix: a virulence determinant of cariogenic biofilm. J Dent Res. 2013;92(12):1065–73. 24045647 10.1177/0022034513504218 PMC3834652

[26] McLean JS. Advancements toward a systems level understanding of the human oral microbiome. Front Cell Infect Microbiol. 2014;4:98. 25120956 10.3389/fcimb.2014.00098 PMC4114298

[27] Cornejo OE, Lefébure T, Pavinski Bitar PD, et al. Evolutionary and population genomics of the cavity causing bacteria Streptococcus mutans. Mol Biol Evol. 2013;30(4):881–93. 23228887 10.1093/molbev/mss278 PMC3603310

[28] Guo L, McLean JS, Lux R, et al. The well-coordinated linkage between acidogenicity and aciduricity via insoluble glucans on the surface of *Streptococcus mutans*. Sci Rep. 2015;5(1):1–11. 10.1038/srep18015 PMC4675080 26657939

[29] Nishimura J, Saito T, Yoneyama H, et al. Biofilm formation by Streptococcus mutans and related bacteria. Adv Microbiol. 2012;2(3):208.

[30] Motegi M, Takagi Y, Yonezawa H, et al. Assessment of genes associated with *Streptococcus mutans* biofilm morphology. Appl Environ Microbiol. 2006;72(9):6277–87. 10.1128/AEM.00614-06. 16957255 10.1128/AEM.00614-06 PMC1563623

[31] Fakhruddin KS, Samaranayake LP, Hamoudi RA, et al. Diversity of site-specific microbes of occlusal and proximal lesions in severe- early childhood caries (S-ECC). J Oral Microbiol. 2022;14(1):2037832. 10.1080/20002297.2022.2037832. 35173909 10.1080/20002297.2022.2037832 PMC8843124

[32] Zhu Y, Wang Y, Zhang S, et al. Association of polymicrobial interactions with dental caries development and prevention. Front Microbiol. 2023;14:1162380. 10.3389/fmicb.2023.1162380. 37275173 10.3389/fmicb.2023.1162380 PMC10232826

[33] Babaeekhou L, Mehrizi AA, Ghane M. *Streptococcus mutans*, sugar consumption, and oral hygiene: which one has more effect on DMFT score in Iranian adults? Dent Res J. 2020;17(2):134. PMC7224268 32435436

[34] Azimi S, Taheri JB, Tennant M, et al. Relationship between mothers’ knowledge and attitude towards the importance of oral health and dental status of their young children. Oral Health Prev Dent. 2018;16(3):265–70. 30027166 10.3290/j.ohpd.a40760

[35] Honda O, Kato C, Kuramitsu HK. Nucleotide sequence of the *Streptococcus mutans* GtfD gene encoding the glucosyltransferase-S enzyme. Microbiology. 1990;136(10):2099–105. 10.1099/00221287-136-10-2099 2148600

[36] Ueda S, Shiroza T, Kuramitsu HK. Sequence analysis of the GtfC gene from *Streptococcus mutans* GS-5. Gene. 1988;69(1):101–9. 2976010 10.1016/0378-1119(88)90382-4

[37] Espinosa-Cristobal L, Martinez-Castanon G, Martinez-Martíinez R, et al. Antimicrobial sensibility of Streptococcus mutans serotypes to silver nanoparticles. Mat Sci Eng C. 2012;32(4):896–901.

