# Supplementary material for: Prevalence of oral streptococci and glucosyltransferase genes in mother-child pairs: a cross-sectional study in Turkish families
Source: BMC Pediatr. 2026 Mar 25;26:330. doi: 10.1186/s12887-025-06479-7 (PMC13085759; doi:10.1186/s12887-025-06479-7)
Supplement: Supplementary file 1 — Supplementary Material 1 [file 12887_2025_6479_MOESM1_ESM.docx]

# **Supplementary Table 1.** Tukey’s Multiple Comparisons Test Results.

| Figure | Group 1 | Group 2 | Comparison Context | Sample Type | P-value | Significance |
| --- | --- | --- | --- | --- | --- | --- |
| Figure 1 | *S. mutans* | *S. sanguinis* | Species distribution | Mother | 0.0185 | * |
| Figure 1 | *S. mutans* | *S. sanguinis* | Species distribution | Child | 0.0055 | ** |
| Figure 1 | *S. mutans* | *S. gordonii* | Species distribution | Child | <0.0001 | **** |
| Figure 2 | *S. mutans* | *S. sanguinis* | Sex-based distribution | Male child | 0.0005 | *** |
| Figure 2 | *S. mutans* | *S. sanguinis* | Sex-based distribution | Female child | 0.5714 | ns |
| Figure 4 | *S. mutans* | *S. oralis* | GTF gene distribution | Child | 0.0018 | ** |
| Figure 4 | *S. salivarius* | *S. oralis* | GTF gene distribution | Mother | 0.0069 | ** |

**Note:** The table summarizes pairwise comparisons performed using Tukey's multiple comparisons test for species distribution, sex-based analysis, and glycosyltransferase gene prevalence. Asterisks indicate levels of statistical significance: *p < 0.05, **p < 0.01, ***p < 0.001, ****p < 0.0001; 'ns' indicates non-significant differences.
