# Supplementary material for: Prevalence of oral streptococci and glucosyltransferase genes in mother-child pairs: a cross-sectional study in Turkish families
Source: BMC Pediatr. 2026 Mar 25;26:330. doi: 10.1186/s12887-025-06479-7 (PMC13085759; doi:10.1186/s12887-025-06479-7)
Supplement: Supplementary file 2 — Supplementary Material 2 [file 12887_2025_6479_MOESM2_ESM.docx]

# **Supplementary Table 2.** Chi-Square Test Results for Categorical Comparisons.

| Comparison Context | Group 1 | Group 2 | Sample Type | Chi-square (χ²) | Degrees of Freedom (df) | P-value | Significance |
| --- | --- | --- | --- | --- | --- | --- | --- |
| *S. mutans* prevalence | Mother | Child | Species comparison | 6.23 | 1 | 0.013 | * |
| *S. gordonii* prevalence | Boy | Girl | Sex-based distribution | 3.89 | 1 | 0.049 | * |
| GtfR gene presence | Mother | Child | GTF gene comparison | 4.11 | 1 | 0.042 | * |
| *S. salivarius* presence | High DMFT | Low DMFT | DMFT-based group | 0.56 | 1 | 0.45 | ns |

**Note:** This table summarizes the results of chi-square (χ²) tests performed to assess associations between categorical variables such as group (mother/child), gender, or caries severity with Streptococcus species prevalence or glycosyltransferase gene presence. A p-value less than 0.05 was considered statistically significant.
